# Synthesis of Heteroaromatic Compounds

**DOI:** 10.3390/molecules28083563

**Published:** 2023-04-19

**Authors:** Joseph Sloop

**Affiliations:** Department of Chemistry, School of Science & Technology, Georgia Gwinnett College, 1000 University Center Lane, Lawrenceville, GA 30043, USA; jsloop@ggc.edu

The synthesis of heteroaromatic compounds has been the subject of intense investigation for well over a century. Studies of the properties exhibited by this broad class of organic molecules have led to countless applications in materials science, agrochemistry and the pharmaceutical industry. This Special Issue, entitled “Synthesis of Heteroaromatic Compounds”, is an outstanding collection of fourteen original research papers and six review articles that discuss the advances made in both conventional and green preparatory methods, as well as the properties and applications of heteroaromatic molecules in industrial and medicinal chemistry areas of study [1,2,3,4,5,6,7,8,9,10,11,12,13,14,15,16,17,18,19,20].

In recent years, computational chemistry methods have been brought to bear to determine both the molecular properties and biomolecular interactions that heteroaromatic compounds exhibit. Molecular docking studies of heteroaromatic compounds have identified myriad potential uses in the treatment of a host of illnesses. A number of the articles found in our Special Issue also leverage this important theoretical tool to allow the reader a better understanding of the impact that new molecules bearing heteroaromatic components may have to both the chemical enterprise writ large and the pharmaceutical industry.

This Special Issue brings together contributions from a truly international and diverse array of ninety experts in the fields of heteroaromatic compound synthesis, QSAR studies, computational chemistry and molecular docking, as well as bioactivity studies. Here are some highlights of the original research work and reviews presented in this Special Issue.

Several articles in this Special Issue feature the synthesis of azole derivatives, including 1,2,4-triazoles; imidazoles; pyrazoles; 1,3,4-oxadiazoles; 1,2,3-triazoles; 1,3,4-thiadiazoles; 1,3-thiazoles; benzimidazoles and isoxazoles. Bollinger and Ardón-Muñoz report a new synthetic protocol to prepare a series of benzo[4,5]thiazolo[2,3-*c*][1,2,4]triazoles derivatives via the C-H bond functionalization of phenyl disulfide intermediates. This method features high functional group tolerance, short reaction times and overall good yields [1]. Kudelko’s group prepared a library of 1,2,4,5-tetrazine-4*H*-1,2,4-triazole conjugates. Compounds of this type may have pharmaceutical uses and possess optoelectronic properties [5]. Saloutin’s team functionalized 2-(polyfluorophenyl)-4*H*-chromen-4-ones via S_N_Ar conditions with 1,2,4-triazole; imidazole; pyrazole and 1,3,4-oxadiazole. Both monoazole and polyazole products were obtained in modest to good yields. Several products demonstrated luminescent properties with potential application as OLEDs; the fungistatic testing of selected products revealed antifungal activity [14]. Kudelko and coworkers prepared a series of novel carboxymethylamino-substituted 2,5-dialkyl-1,3,4-oxadiazoles via a multistep method employing commercially available acid chlorides, hydrazine hydrate and phosphorus oxychloride in yields as high as 91% [9]. Gribanov’s team prepared new examples of 5-(het)arylamino-1,2,3-triazole derivatives in high yields via a palladium-complex-catalyzed Buchwald–Hartwig cross-coupling reaction of 5-amino- or 5-halo-1,2,3-triazoles with (het)aryl halides and amines [2]. Obydennov and coworkers synthesized a new class of conjugated pyrans based on the examination of 2-methyl-4-pyrones with DMF-DMA, including derivatives bearing isoxazole and benzimidazole ring systems. The compounds demonstrated valuable photophysical properties, such as large Stokes shifts and good quantum yield [12]. Sosnovskikh’s group synthesized a series of 3-hydroxy-3,4-dihydropyrido[2,1-*c*][1,4]oxazine-1,8-diones, which are novel building blocks for biologically important polycyclic pyridones via an oxazinone ring-opening transformation. The partial aromatization of the heterocycles formed polycyclic benzimidazole-fused pyridines [15]. Fedetov’s team prepared a series of novel 4-(aryl)-benzo[4,5]imidazo[1,2-*a*]pyrimidine-3-carbonitriles via Povarov and oxidation reactions from benzimidazole-2-arylimines. The photophysical properties of the studied compounds included positive emission solvatochromism with large Stokes shifts [10]. Nakano and coworkers designed spiro[indeno[1,2-*b*][1]benzofuran-10,10′-indeno[1,2-*b*][1]benzothiophene] as well as the *S*,*S*-dioxide derivative and the pyrrole-containing analog. The furan-containing chiral spiro-fused PACs were found to be circularly polarized luminescent materials [7]. Guerrini’s team prepared a series of 5-oxo-4,5-dihydropyrazolo[1,5-a]thieno[2,3-e]pyrimidine derivatives as potential GABA_A_ modulators. These compounds were studied using the ‘Proximity Frequency’ model with molecular docking and dynamic simulation; all products were determined to have an agonist profile, highlighting the suitability of the nucleus to interact with the receptor protein [18].

Several papers within this collection not only feature the synthesis of new heteroaromatic compounds but also investigate the potential biological applications of those molecules. Xiang’s group prepared a series of 8*H*-indeno[1,2-*d*]thiazole derivatives and evaluated their inhibitory activities against SARS-CoV-2 3CL^pro^ through a high-throughput screening of their compound collection. One compound was identified as a novel SARS-CoV-2 3CL^pro^ inhibitor and was subjected to molecular docking to predict the binding mode with SARS-CoV-2 3CL^pro^ [4]. Gomha’s group synthesized 3-aryl-5-substituted 1,3,4-thiadiazoles, 3-phenyl-4-arylthiazoles and the 4-methyl-3-phenyl-5-substituted thiazoles from 1-(3-cyano-4,6-dimethyl-2-oxopyridin-1(2*H*)-yl)-3-phenylthiourea and hydrazonoyl halides, α-haloketones, 3-chloropentane-2,4-dione and ethyl 2-chloro-3-oxobutanoate. The new compounds showed anticancer activity against the cell line of human colon carcinoma (HTC-116) as well as hepatocellular carcinoma (HepG-2). Molecular docking studies of the thiadiazol confirmed a binding site with EGFR TK [8]. Another team under Gomha’s direction designed and synthesized novel 3-thiazolhydrazinylcoumarins via the reaction of phenylazoacetylcoumarin with various hydrazonoyl halides and α-bromoketones. Molecular docking studies of the resulting 6-(phenyldiazenyl)-2*H*-chromen-2-one derivatives were assessed against VEGFR-2 and demonstrated comparable activities to that of Sorafenib (an approved medicine). The cytotoxicity of the most active thiazole derivatives was investigated for their efficacy against human breast cancer (MCF-7) cell line and normal cell line LLC-Mk2 using an MTT assay and Sorafenib as the reference drug. Several compounds were found to have higher anticancer activities than Sorafenib [13]. Yang’s group prepared a series of new *N*-(thiophen-2-yl) nicotinamide derivatives via the nucleophilic acyl substitution of nicotinic acid chlorides and aminothiophenes. The in vivo bioassay results of all the compounds against cucumber downy mildew (CDM; *Pseudoperonospora cubensis*) indicated that several compounds exhibited fungicidal activities higher than both diflumetorim and flumorph fungicides [11].

This Special Issue also features reviews of topics that are of ongoing interest to the scientific community in the areas of heteroaromatic synthesis and applications. Henary and Tran reviewed the synthesis and applications of antiviral agents derived from various nitrogen-containing heteroaromatic moieties, such as indole, pyrrole, pyrimidine, pyrazole and quinoline, within the last decade. The synthesized scaffolds target HIV, HCV/HBV, VZV/HSV, SARS-CoV, COVID-19 and influenza [3]. Schoffstall and Hoffman examined the current state of synthesis methodologies used to prepare pyrazines and pyridazines fused to 1,2,3-triazoles. The review also details the use of these heterocycles in medicinal chemistry as c-Met inhibitors or GABA_A_ modulators, in materials science as fluorescent probes, and as structural units of polymers [6]. Murai and Hashimoto give a comprehensive accounting of both conventional and green synthetic methodologies used to prepare (3-trifluoromethyl)diazirine-substituted heteroaromatics, including pyrimidines, pyridines, benzimidazoles, pyrazoles, benzoxazoles, benzothiophenes and indoles. The authors also highlight medicinal, polymer and materials science applications for these diazirine-substituted heteroaromatics [16]. Rogalski and Pietraszuk explore recent advances in the application of the olefin metathesis reaction, particularly the cyclization of dienes and enynes, in synthesis protocols leading to (hetero)aromatic compounds, including pyrroles, furans, indolizines, benzofurans, pyrimidiniums, indoles, pyridoindoles and carbazoles. Several examples of green preparations of these heteroaromatic compounds are described [17]. Asquith’s group provided a thorough review of the synthesis methodologies used to prepare 1,2,3-dithiazoles, their reactivity with other substrates, and their medicinal uses as antifungals, herbicides, antibacterials, anticancer agents, antivirals and antifibrotics, and as melanin and Arabidopsis gibberellin 2-oxidase inhibitors [19]. In their review of heteroaromatic hybrid chalcones, Mallia and Sloop outline the recent advances made in the incorporation of heteroaromatic moieties in the synthesis of hybrid chalcones. Examples of environmentally responsible processes employed in the preparation of this important class of organic compound are also highlighted [20].
